# End-to-End Solution for Analog Gauge Monitoring Using Computer Vision in an IoT Platform

**DOI:** 10.3390/s23249858

**Published:** 2023-12-15

**Authors:** João Peixoto, João Sousa, Ricardo Carvalho, Gonçalo Santos, Ricardo Cardoso, Ana Reis

**Affiliations:** 1 Faculty of Engineering, University of Porto, 4200-465 Porto, Portugal; jpsousa@inegi.up.pt (J.S.); areis@inegi.up.pt (A.R.); 2 INEGI—Institute of Science and Innovation in Mechanical and Industrial Engineering, 4200-465 Porto, Portugal; rjcarvalho@inegi.up.pt (R.C.); rcardoso@inegi.up.pt (R.C.); 3 Glarevision S.A., 2400-441 Leiria, Portugal; goncalo.santos@glartek.com

**Keywords:** computer vision, cloud computing, IoT, remote monitoring

## Abstract

The emergence of Industry 4.0 and 5.0 technologies has enabled the digital transformation of various processes and the integration of sensors with the internet. Despite these strides, many industrial sectors still rely on visual inspection of physical processes, especially those employing analog gauges. This method of monitoring introduces the risk of human errors and inefficiencies. Automating these processes has the potential, not only to boost productivity for companies, but also potentially reduce risks for workers. Therefore, this paper proposes an end-to-end solution to digitize analog gauges and monitor them using computer vision through integrating them into an IoT architecture, to tackle these problems. Our prototype device has been designed to capture images of gauges and transmit them to a remote server, where computer vision algorithms analyze the images and obtain gauge readings. These algorithms achieved adequate robustness and accuracy for industrial environments, with an average relative error of 0.95%. In addition, the gauge data were seamlessly integrated into an IoT platform leveraging computer vision and cloud computing technologies. This integration empowers users to create custom dashboards for real-time gauge monitoring, while also enabling them to set thresholds, alarms, and warnings, as needed. The proposed solution was tested and validated in a real-world industrial scenario, demonstrating the solution’s potential to be implemented in a large-scale setting to serve workers, reduce costs, and increase productivity.

## 1. Introduction

The manufacturing industry is undergoing rapid and radical transformations as it embraces the latest technological advances. From Industry 4.0 to 5.0, the sector is witnessing a shift towards greater connectivity, intelligence, digitization, and automation of its processes and systems [1,2]. These changes aim to enhance productivity, efficiency, quality, and sustainability, as well as to foster a more human-centric and collaborative work environment for shop floor workers [3].

Despite the manufacturing industry being one of the early adopters of these technologies, it still faces significant challenges in terms of implementation and adoption [4,5]. While in some areas companies have successfully integrated advanced technologies such as the Internet of Things (IoT) [6,7], artificial intelligence (AI) [8], and robotics [9] into their operations, others have struggled to keep up [10,11]. In various industries such as oil and gas refineries [12,13], chemicals [14], and manufacturing [15,16], numerous physical processes require monitoring of values like voltage, current, pressure, and temperature. Often, the monitoring of these parameters relies on analog gauges, with readings manually recorded by shop floor workers. However, the conventional method of using analog gauges for monitoring presents a series of challenges. These gauges are typically installed in environments that are distant, harsh, or otherwise inaccessible, making it difficult for workers to consistently and accurately collect data. The physical constraints of these environments not only pose challenges to the workforce but also impact the overall efficiency, quality, and profitability of industrial operations. The limitations of manual monitoring become particularly evident in industries where real-time data are crucial for decision-making. In sectors like oil and gas, where precision and timeliness are imperative for safety and production optimization, the reliance on traditional gauges can hinder the ability to respond promptly to changes or anomalies in processes [2]. This delay in data acquisition and response time may lead to inefficiencies, compromised product quality, and, in extreme cases, safety risks. Therefore, a system to tackle this would not only enhance the accuracy and immediacy of data collection but also mitigate the risks associated with manual readings in challenging environments.

The current paper proposes an end-to-end solution to digitize analog gauges and monitor them to help shop floor operators and maintenance teams. Therefore, a prototype monitoring device was designed to accommodate a camera to capture images in a controlled environment with homogeneous illumination, in order to facilitate the computer vision algorithm in identifying the gauge. Additionally, a platform was developed to facilitate operators in monitoring industrial gauges and give them the flexibility to create thresholds, alarms, and warnings for each monitored gauge. This solution stands out from other approaches due to its exceptional robustness. Unlike many research endeavors that focused on a limited set of gauges and were primarily tested in controlled environments, our approach is uniquely designed to accommodate a diverse range of analog gauges in terms of types and sizes. Furthermore, our solution has an artificially controlled environment using an enclosed device, specifically tailored for the demanding conditions of industrial applications. This controlled environment ensures the robustness and reliability of our approach, making it well-suited for harsh industrial settings.

This article is organized into four sections. Section 1 is the introduction, where the context is explained, and in Section 2 state-of-the-art works are analyzed. Section 3 provides the proposed IoT gauge monitoring platform and details the proposed solution, in terms of the overall architecture, device design, computer vision algorithm, and IoT platform for implementation. Then, in Section 4, the solution is tested and validated in a real-world scenario to emulate a use case. Finally, in Section 5, the study concludes with a comprehensive discussion of the work carried out and highlights the strengths and limitations.

## 2. Background and Related Work

In many diverse industries, IoT devices are already being applied to monitor processes with cloud computing architectures, which are rapidly evolving and gaining popularity. Cloud computing enables companies to store, manage, and analyze the large amounts of data generated by IoT devices, making it an ideal solution for industrial applications [17,18]. Real-time monitoring and automated data analysis in manufacturing enterprises can provide product and process tracking, as well as predictive maintenance that is achieved using machine learning (ML) [19] or deep learning (DL) techniques [20], through finding patterns and anomalies in the data. This offers a wide range of benefits, including increased efficiency, improved safety, and reduced costs [21].

A real application of this technology is in the predictive maintenance of industrial equipment, which has become a critical aspect in Industry 4.0; for example, Magadán et al. [22] proposed a system to monitor electric motors in real-time for the detection of operating anomalies and predictive maintenance. The design used low-cost hardware components, open-source software, and an IoT analytics service in the cloud, where the information was stored.

Since analog gauges are ubiquitous in industrial settings, providing real-time information about various parameters such as pressure, temperature, and flow rate, they serve as an excellent case study for these technologies, to digitize and monitor them. This is especially true because manually reading analog gauges can be time-consuming, prone to human error, and hazardous in certain environments. To address these challenges, researchers and companies have explored the use of computer vision (CV) and IoT devices for automated analog gauge reading. For example, the company Lilz developed a device that integrates computer vision and IoT to automate the reading of analog gauges. By placing a camera in a fixed position relative to the gauge, operators can precisely define the gauge’s location and key points. This technology not only automates the reading process but also monitors the gauge through an integrated IoT platform, allowing industrial parameter monitoring.

For gauge monitoring, there are various solutions based on computer vision, which can be categorized into traditional approaches and neural network (NN) approaches. The most common NN models used are object detection models for identifying gauges in images and optical character recognition (OCR) for extracting the dial values [23,24,25]. However, these models, despite being trained on extensive datasets, often suffer from reliability issues in industrial settings. This is primarily due to the poor condition of the gauges, inadequate illumination, and harsh environmental factors [26]. On the contrary, the traditional computer vision techniques often work well in noisy and complex environments, where NN-based techniques may struggle to generalize, and they are often less computationally intensive [27,28]. Moreover, several authors have developed algorithms only using traditional computer vision techniques that are robust and accurate, for example, Gellaboina et al. [29] proposed an algorithm that can automatically identify dial gauge readings using an image captured by a handheld device. This was tested on multiple dial gauge images captured in a local chiller plant, with reliable readings in up to 95% of cases. In addition, Tran et al. [30] developed a system to read analog gauges at a power substation that used computer vision techniques, such as color segmentation [31], Canny edge detection [32], and Hough circle transform [33] to detect the pointer and the scale marks of gauges. However, these systems are still mostly limited to the tested gauges, and the accuracy obtained was only for very controlled environments and limited situations.

Therefore, this article presents a robust solution for monitoring different gauges in harsh industrial environments. The proposed solution includes a monitoring device composed of an outer case that safeguards essential components like the camera, PCB, and LEDs. This solution helps to homogenize the images captured to facilitate the automation of the gauge reading process using computer vision techniques. To facilitate remote monitoring of field and shop floor gauges, the solution was integrated into an Internet of things (IoT) architecture. This integration allows companies, operators, and workers to conveniently monitor the gauges from a remote location. By leveraging computer vision and IoT technologies, this solution offers a robust and efficient approach to gauge monitoring in challenging industrial settings.

## 3. Proposed IoT Gauge Monitoring Platform

The proposed solution for an IoT gauge monitoring platform is composed of a physical device to monitor gauges by capturing images in a controlled environment that is integrated into an IoT architecture using cloud computing, which is also responsible for executing the computer vision algorithm capable of reading analog gauges. Thus, this section starts by presenting the overall architecture of the IoT gauge monitoring platform, followed by a detailed overview of the monitoring device and the computer vision algorithm. In the end, the IoT platform is explained, which includes the interface through to the end user.

### 3.1. Architecture

**Figure 1:**
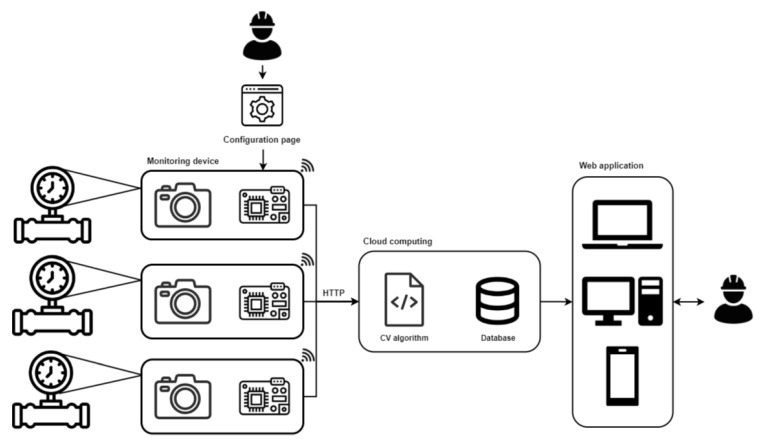
Overall architecture of the IoT gauge monitoring platform.

The overall architecture of the IoT platform is illustrated in Figure 1, and this includes the aforementioned monitoring device that needs to be coupled with an analog gauge, which needs to fit different sizes and types of gauges and withstand the adverse environments that are characteristic of a wide range of industries. As the device is installed within a gauge and designed to adapt to various gauge sizes for enhanced flexibility, the operator can access a web-based interface for configuring the device. Through this interface, the operator can define the specific characteristics of the currently utilized gauge and set parameters such as the intervals for capturing images or configuring connection credentials. The monitoring device includes a camera sensor and microcontroller for capturing gauge images and sending them to the cloud. The computer vision algorithm processes these images to read gauge values, which are stored in a database. Users access these data via a web-based interface, allowing for customized data presentation, warnings, and alarms.

### 3.2. Monitoring Device

As mentioned before, the proposed IoT gauge monitoring platform requires the utilization of a microcontroller-controlled camera for capturing periodic images. The images are enhanced using LED lighting to control the environmental illumination. Additionally, the platform includes a battery for power supply and a Wi-Fi antenna to establish a wireless connection. To ensure the safety and functionality of the device, we designed an affordable and user-friendly outer case, specifically for easy installation on various sizes of industrial gauges. This case offers both protection and accessibility, enabling maintenance operations, addressing unexpected malfunctions, and facilitating responses to emergency situations.

**Figure 2:**
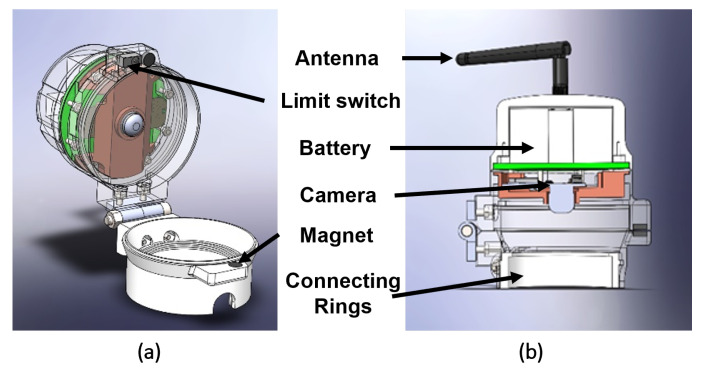
Design of the outer case using CAD. (**a**) Case in the open position. (**b**) Case closed and with a middle section plane.

The outer case design is represented in Figure 2 and takes into consideration all the components that should be sheltered, protecting them from the industrial environment. The design of the case is intended to give the users intuitive interactions in a simple and compact structure. The case has a circular base with a connecting ring, which enables the adaptation of the device to different sizes of circular gauges. The case can be opened to access the gauge, as well as closed and locked using magnets. The upper part has the camera, LEDs, and the main microcontroller connected to a battery as the power source. We have also incorporated an antenna, to obtain a reliable wireless connection to send the captured images to the cloud. Additionally, a limit switch is installed, to determine if the case is open, which has a specific function for the configuration of the microcontroller, as explored in more detail in Section 3.3.

**Figure 3:**
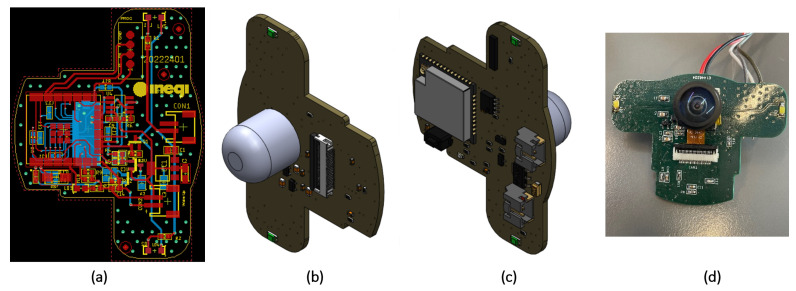
Conception of PCB based on ESP32. (**a**) PCB routing. (**b**) Front view of the PCB. (**c**) Rear view of the PCB. (**d**) Real image of the PCB.

To house the microcontroller, camera sensor, and LEDs, a specific PCB was designed based on the ESP32-CAM from Espressif Systems, Shanghai, China, with a compact and easy-to-install geometry. Two LEDs were also added to illuminate the analog gauge, helping create a homogeneous environment. In addition, the components from the original ESP32-CAM that had no function in this device were removed. Figure 3 shows the PCB design and the final version printed. It is important to note that the LEDs are pointed directly at a diffuser acrylic disk, and because the ESP-32 controls the LED brightness, it is possible to maintain light uniformity without any reflections inside the case.

**Figure 4:**
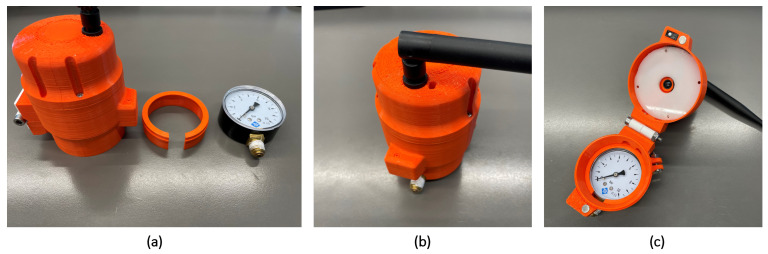
Mounting the monitoring device. (**a**) Unmounted monitoring device. (**b**) Closed monitoring device. (**c**) Open monitoring device.

The final case design was printed in polylactic acid (PLA) in a 3D printer, then the connecting ring and the components were installed inside and the case was fixed with screws. As exemplified in Figure 4, the device was mounted by first connecting the correct size ring with the case and then attaching it to the gauge and tightening the locking screw to fix the device.

### 3.3. Computer Vision Algorithm

The algorithm employed for the interpretation of analog gauges represents a modified version of a code initially introduced in a prior publication [25]. We made some modifications to tailor it for compatibility with the monitoring device. The primary goal was to enhance its robustness, accuracy, and resilience in an industrial setting. The present solution provides a distinct advantage by operating within a controlled environment. This not only simplifies gauge reading but also allows for the utilization of a more efficient computational algorithm, reducing computational overheads.

**Figure 5:**
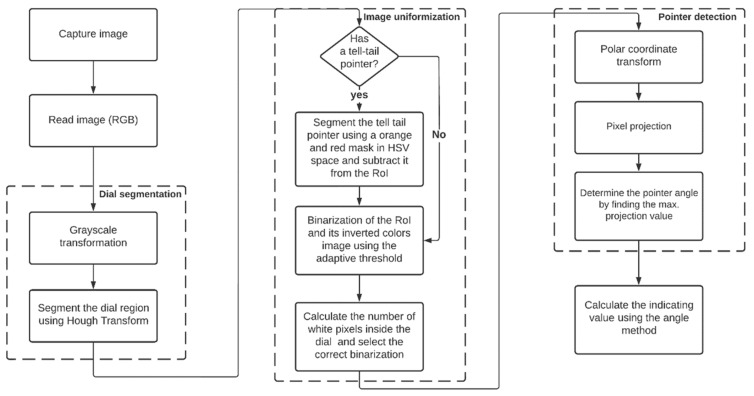
Flowchart of the steps used to read the analog gauges.

**Figure 6:**
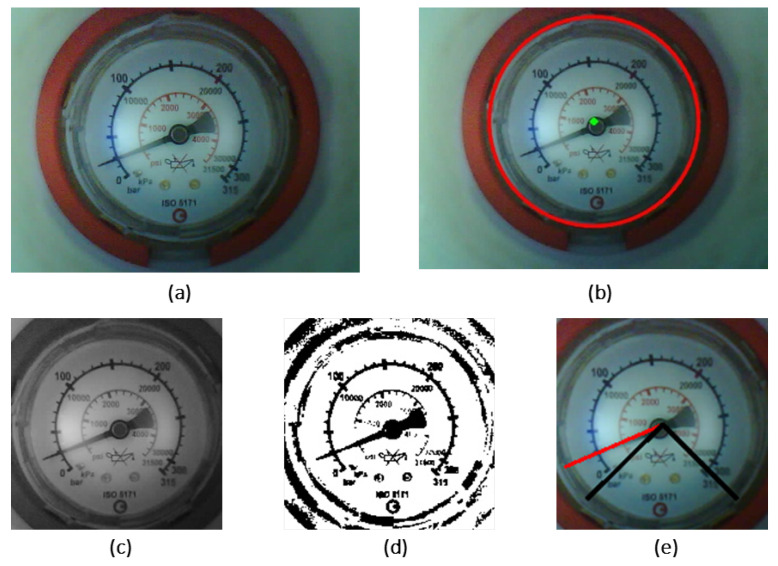
Main steps of the gauge-reading algorithm. (**a**) Original image. (**b**) Gauge dial detection. (**c**) Cropped grayscale image. (**d**) Binarized image. (**e**) Gauge pointer detection.

The proposed computer vision solution for automatic gauge reading consists of several key steps, as illustrated in Figure 5. The process begins with the microcontroller capturing an image that is subsequently sent to the cloud for further processing. The image is first converted into grayscale and the dial is detected using circle Hough transformation, and with this technique the circle radius and center are extracted. Since there are a lot of variations among analog gauges, the images are uniformized for gauges with different dial colors (white and black) and the presence of tell-tail pointers. Since the tell-tail pointer is usually orange or red, to extract their values and uniformize the original image, a mask for these colors in HSV color space is used to segment and crop that region from the original image, to then detect the main pointer using the cropped image. Afterward, a polar coordinate transformation is performed, followed by a pixel projection to detect the pointer angle by finding the highest projected value. In the end, the gauge value is calculated using the angle method. Figure 6 shows images from the execution of the algorithm during image capture using the capturing device.

**Figure 7:**
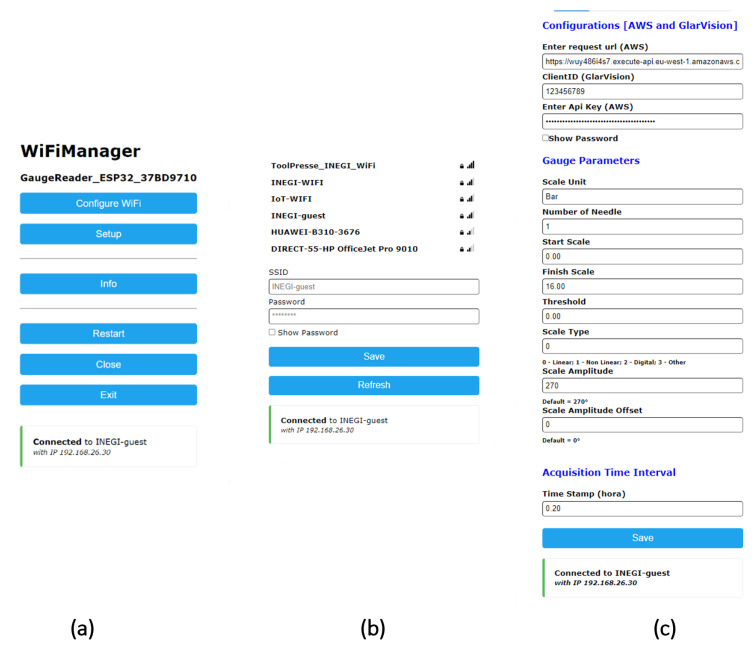
Web page for microcontroller configuration. (**a**) Configuration main page. (**b**) Wi-Fi configuration. (**c**) Gauge parameter configuration.

In order to calculate the indicated gauge value, data related to the gauge scale are required, so this information is incorporated into the metadata in each image, to be decoded later when the gauge reading process in the cloud is executed. Since the device monitors just one gauge, we developed a configuration page to define several variables related to the gauge being monitored for each microcontroller, as detailed in Table 1. The configuration web page, as depicted in Figure 7, serves as a user-friendly platform for setting up the microcontroller to read a specific gauge. The user has the capability to configure the Wi-Fi connection, set gauge parameters as previously discussed, input necessary credentials for secure cloud access, and specify the preferred time intervals for image capture. These settings accommodate diverse application needs. Furthermore, the microcontroller’s configuration can be activated by opening the device’s case, facilitated by the inclusion of a limit switch.

### 3.4. IoT Platform

**Figure 8:**
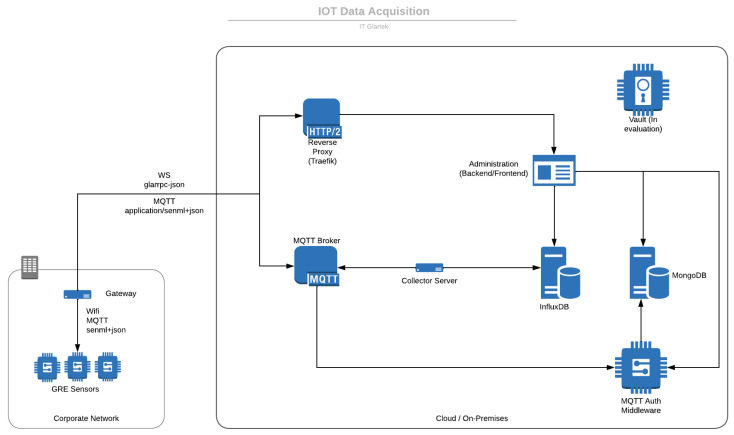
High lever architecture implemented.

Then, an IoT platform was developed to serve as an interface between users and industrial devices. The platform has been designed to establish reliable communication with a wide range of IoT devices while maintaining a high level of security. The platform architecture, as represented in Figure 8, consists of four layers: devices, edge computing, cloud computing, and applications.

The devices layer includes our gauge reader, which sends data to the edge computing layer. Here, the data are processed, and tasks such as executing the computer vision algorithm to extract the gauge values from images sent by the device are performed. The cloud computing layer offers storage, processing, and analytics capabilities to manage the data generated by the edge computing layer. Finally, the applications layer provides end-users with the ability to analyze data and take necessary action to improve the processes.

Since multiple devices are intended to collect data, there is a need to store the information in a secure and protected environment. Thus, the collector server service validates the received data, converting it into a format that can be inserted into the database, and inserting the received data into the time series database, which in this case InfluxDB was used. In addition, the interface developed for the user allows the configuration of new sensors on the platform, which makes it possible to create a new MQTT account using the cloud module MQTT Auth Middleware. Therefore, the developed system can generate a new device type and create specific credentials for them. The security of the IIoT system was ensured using robust security measures, such as data encryption, access control, and authentication.

MQTT was chosen because it is a lightweight and efficient protocol designed for low-bandwidth, high-latency, or unreliable networks, which makes it well-suited for IoT applications. Although the protocol has security risks and vulnerabilities, we mitigated these with additional security measures. This includes the encryption of the communication between IoT devices and servers to help prevent unauthorized access to sensitive data and protect against man-in-the-middle attacks. Additionally, access control lists (ACLs) are employed to restrict access to specific topics and resources, preventing unauthorized clients from subscribing or publishing to sensitive topics, while regular security audits and vulnerability assessments are conducted to identify and address any emerging security issues promptly. In order for this kind of architecture to be differentiated from others and avoid enterprise network restrictions, we have used the ALPN extensions from the TLS protocol. TLS-ALPN allows for the negotiation of multiple protocols within a single TLS connection, which is useful in IoT solutions, where multiple protocols may be used to communicate with the same server, making this solution flexible. This means that an IoT device can communicate with a server using both HTTPS and MQTT protocols using the same port. By negotiating multiple protocols within a single TLS connection, the resources on IoT devices are conserved, which often have limited processing power and memory.

In summary, the IoT platform developed offers a seamless solution for users to interact with their industrial devices. It was designed to be secure, flexible, and scalable, to improve overall industrial processes by increasing efficiency and productivity.

## 4. Tests and Validation

**Figure 9:**
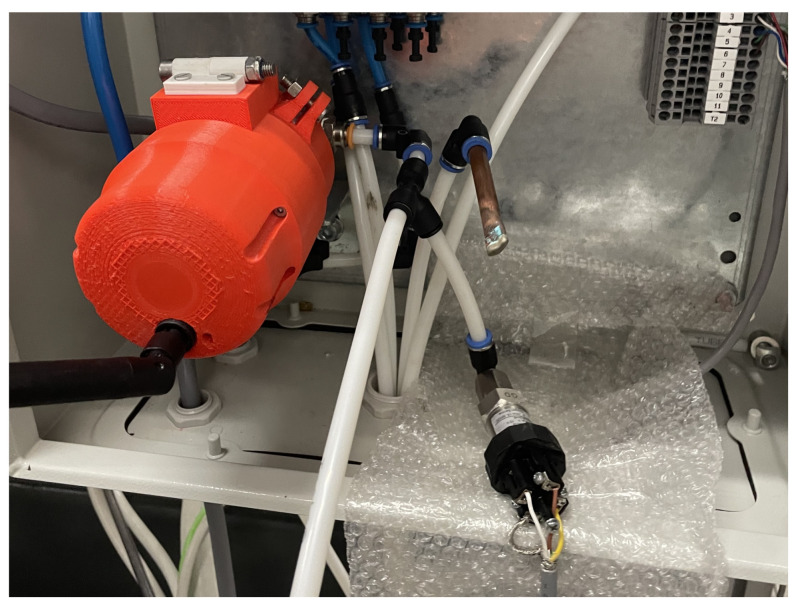
Setup of the monitoring device test, with the monitoring device and a transducer.

The IoT platform was finally tested at INEGI’s industrial facilities. This process began by installing the monitoring device within a pressure gauge responsible for monitoring the air pressure supplied to a laser cutting machine. The monitoring device was mounted, as shown in Figure 9, with a pressure transducer on the side. The pressure of the feeding line was controlled manually through a pressure valve and it was changed between 2.5 and 7 Bar. Prior to commencing the test, the device was properly configured with the necessary information specific to the selected gauge, to ensure accurate readings.

**Figure 10:**
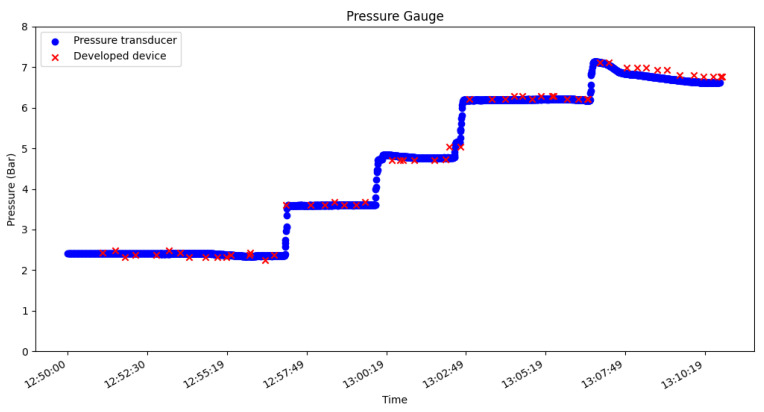
Test of the pressure gauge from 7 to 2.5 Bar.

The data was saved including the images captured, and a graph was drawn as shown in Figure 9, which shows the gas pressure during the testing period. The pressure values read by the monitoring device were monitored using a pressure transducer, to corroborate the values obtained by the device. As depicted in Figure 10, the values exhibited slight fluctuations, which can be attributed to the gauge’s vibration and its pointer, in conjunction with the error of the computer vision algorithm. In this test, the maximum absolute error was 0.217 Bar, which corresponds to a relative error of 1.81%.

**Figure 11:**
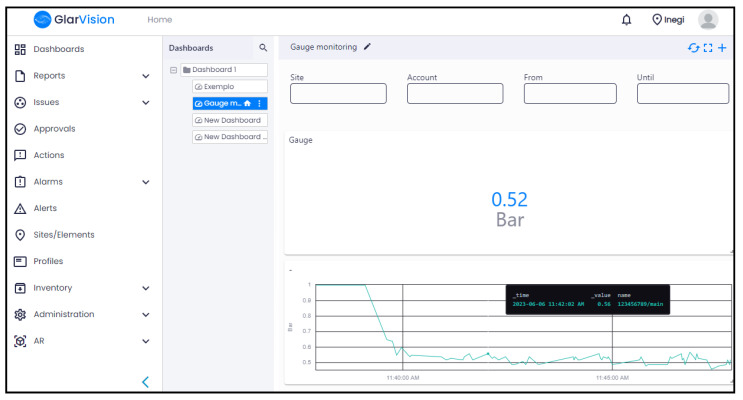
Example of a dashboard in the IoT platform.

As illustrated in Figure 11, a dashboard was created in the IoT platform with two widgets, which include a line graph and a value to be used by the operators to remotely monitor them.

## 5. Discussion and Future Work

Initially, we designed a protective outer case capable of housing the PCB, camera, LEDs for light control, battery, and connecting rings to adapt to different gauge sizes. The case was designed to provide direct access to the gauge and allow interaction, if required by the operator. Additionally, the open position of the case enables the PCB to be put into configuration mode, allowing the user to define and modify the gauge parameters. This case not only facilitates control over critical image conditions such as illumination for computer vision algorithms but also protects the components from the harsh industrial environment. The gauge values are continuously monitored by the device using a computer vision algorithm that, combined with the gauge parameters defined by the user, can determine the gauge features and determine the indicated value. Afterward, an IoT platform was developed to show the data to the users. This was designed to be reliable and flexible to the user’s needs, and it lets the user develop their own dashboards.

To pave the way for the widespread adoption of this monitoring device, a series of essential steps lie ahead. These encompass rigorous research and development efforts aimed at refining the device’s design, reducing production costs, and enhancing its overall resilience. Concurrently, comprehensive testing protocols must be undertaken to ensure compliance with country-specific regulations, spanning criteria like battery capacity, WiFi coverage, and resistance to electromagnetic interference, particularly in the demanding industrial settings characterized by dusty environments.

In our ongoing efforts to enhance the monitoring device, future research will be dedicated to refining its design, reducing production costs, and improving overall resilience. We are exploring alternative manufacturing methods, with a particular emphasis on injection molding, to address the constraints associated with additive manufacturing. This diversification aims to identify cost-effective and scalable options for mass production. Adapting our system to various pressure gauges has proven challenging, due to the diverse range of types, shapes, and configurations, and compounded by the impact of humidity and vibrations on the vision system. Our forthcoming focus will concentrate on developing a more versatile and adaptable design, simplifying the adaptation process and allowing seamless adjustments for different pressure gauge specifications. This approach aims to significantly enhance the device’s overall applicability. While Wi-Fi connectivity offers versatility, its limitations in factory environments have prompted us to explore alternatives such as power over Ethernet (PoE). Thus, we are considering developing dual versions of the device, one with Wi-Fi and another with PoE, to provide users with flexibility based on their specific operational needs. Similarly, it would be beneficial to have power source flexibility, including options for electrical grid connections, batteries, or cells, which is essential to address distinct application needs effectively. These would provide users with choices that align with their specific requirements. Additionally, this iterative process will involve rigorous testing, feedback collection, and collaboration, so partnering with industrial companies is important, especially to ensure that the device meets the highest standards of performance and reliability.

## 6. Conclusions

The objective of this study was to create a comprehensive solution capable of acquiring real-time data from analog gauges, aiming to tackle the challenges encountered in industries like oil and gas, where manual monitoring of physical processes with these instruments remains a common practice. To accomplish this, a monitoring device was designed to continuously track the gauge values using a computer vision algorithm. This was achieved by combining user-defined gauge parameters with a computer vision algorithm, which determines the gauge features and the corresponding indicated value. Subsequently, we developed an IoT platform to present these data to the users, allowing users to create their own customized dashboards according to their specific needs.

In conclusion, the work undertaken in this study has made significant contributions to our understanding of the prototype device’s practical application in real industrial settings, highlighting its potential as a valuable solution. Furthermore, we successfully demonstrated the seamless integration of this device into an IoT cloud platform, showcasing its adaptability and scalability within the digital ecosystem. Our efforts confirmed the prototype device’s viability in industrial contexts and highlighted its pivotal role in the evolving IoT landscape, serving as both a testament to our achievements and a road map for its continued development and implementation in the industrial sector.

## Figures and Tables

**Table 1:** Metadata of the images.

Metadata	Description
NrNeedles	Number of pointers
StartScale	Start scale angle
FinishScale	End scale angle
TypeScale	Type of scale (Ex: linear)
Threshold	Limit of gauge value to send warnings
ScaleAmplitude	Angular amplitude of scale
ScaleAmplitudeOffset	Offset of the scale amplitude
TimeStamp	Timestamp of the captured image

## Data Availability

Data are contained within the manuscript.

## Abbreviations

The following abbreviations are used in this manuscript:

IoT	Internet of Things
AI	Artificial Intelligence
LED	Light Emitting Diode
PCB	Printed Circuit Board
DL	Deep Learning
ML	Machine Learning
NN	Neural Network
OCR	Optical Character Recognition
